# Mature Australian VET markets: a data-driven case study of public policy implementation

**DOI:** 10.1186/s40461-022-00133-7

**Published:** 2022-05-06

**Authors:** Don Zoellner

**Affiliations:** Northern Institute, Charles Darwin University, Grevillea Drive, Alice Springs, NT Australia

**Keywords:** VET markets, Policy implementation, Data, Policy analysis

## Abstract

Australian vocational education and training (VET) policy makers have persistently proposed more user choice when reforming the national training system. Increasing alternatives by encouraging multiple providers to trade in regulated contestable markets remains the cornerstone of governments’ policies. However, despite policy intentions, students’ options are declining. Longitudinal quantitative jurisdiction-level statistics identify well-established trends of a reduced variety of providers, a smaller range of qualifications on offer and decreased public funding. These outcomes are occurring notwithstanding the continuous supportive policy environment for intensified competition and amplified choice. Rather than portray reduced choice as policy failure, this research makes novel use of large nationally consistent regulatory and enrolment data sets to provide evidence of successful public policy implementation that is approaching the end of the market life cycle. The results invite an exploration of issues that arise when VET markets are considered to be mature rather than unrealised aspirations. Choices provided in these fully-fledged markets that balance public and private provision are still necessary, but no longer sufficient, to meet national skills needs. It is argued that policy success is not permanent and should be revisited in light of contemporary economic alternatives to guide future VET policy making.

## Introduction

In the late 1980s the Australian Government indicated that it planned to partner with states and territories to improve the relevance of their Technical and Further Education (TAFE) institutions. Anticipated benefits included the creation of a federated training system to meet national objectives, utilising existing resources to improve productivity, achieving efficiency gains and producing ‘further increases in participation in vocational education and training, [including] an expansion of total training capacity’ (Dawkins and Holding 1987, 34). Special ministerial conferences in 1989 and 1990 agreed to expand VET offerings by creating ‘an open national training market’ as one element of the National Training Reform Agenda (Lundberg 1994, 1).

International agencies such as the OECD actively encouraged governments to introduce markets and New Public Management (NPM) as a mechanism of policy implementation in the 1980s. NPM allowed governments to procure services by contracting private and public agents to implement policies, achieve specified outputs and operate in an effective regulatory environment (Hill and Hupe 2002, 110). By adopting NPM, the responsibility for policy failure is contracted away and the role of government shifts from program delivery to contract management and regulation. Former senior federal government bureaucrat, Keating (2004, 12) notes Australia has experienced bi-partisan endorsement of NPM due to ‘the attractiveness of markets as a policy instrument for governments’.

Bowman and McKenna (2016, 16) report that the objective of ‘an effective training market with public and private providers’ has appeared in ‘all subsequent national VET agreements’ between state, territory and federal governments since 1992. Repetitions of the 1980s position often characterise open markets as a yet to be obtained goal or policy innovation (for example, Productivity Commission 2020, 2). Nevertheless, contestable VET markets were successfully established and continue to offer choice to individual students as well as being used to procure government services from a diverse range of providers.

Peters (2021, 2) describes Australia as one of three countries considered to be important and successful ‘global pioneers’ in the introduction of markets for the economic sales of vocational training. This article makes innovative use of quantitative data extracted from the large statistical collections of organisations that regulate different portions of the Australian VET market to analyse and interpret how this international ‘first mover’ positioning has played out over the past 30 years. Three related research questions are addressed:

- What have been the realised benefits of VET marketization?
- Is the market still achieving the original policy intention?
- Has time taken a toll resulting in counter-productive outcomes?

This research project has been guided by a theoretical framing of the corporate (and related market) life cycle. The five stages are not always linear and observed in every case, but generally progress through birth, growth, maturity, revival and decline (Miller and Friesen 1984, 1161). Particular attention will be paid to the characteristics of the maturity phase given the longstanding public policy consistency in favour of contestable markets and increased user choice. If it is determined that Australian VET markets are in decline and no longer meeting the original policy intentions, some emerging alternatives based on public value and system optimisation are suggested.

## Background

The ‘normative conception of a single national training market, with credentials articulated in a common system, was adopted by all Australian governments in November 1990 as the framework for modernisation reform’ when they accepted the OECD-led global policy consensus of the time in favour of open and competitive training markets (Marginson 1997, 270–271). The initial open marketplace design had low barriers to entry allowing an expansive range of VET providers to deliver national qualifications. Included were:

TAFE colleges, higher education, private training providers including business colleges, correspondence schools and private tertiary institutions, community and voluntary groups, employers offering training to their own employees and other organisations on a host basis, professional organisations and Industry Training Advisory Bodies (Marginson 1997, 271).

The first national VET strategy reaffirmed this policy decision. ‘The key to making vocational education and training responsive is to enhance the choice that clients have between the full range of providers—public, private and industry’ (Australian National Training Authority 1994, 7). This strategic objective required state and territory governments to eliminate their TAFE monopolies by allowing other providers access to public funding through competitive tendering (Deveson 1990, 26).

These governments had agreed that the lack of choice of VET provider was a problem. Fixing the problem required that the issue be expressed in a manner that is amenable to resolution within the limits of government authority and capacity (Bacchi 2009, viii–ix). In their joint desire to establish a National Training System, the state, territory and federal governments problematised the absence of choice of training providers competing in an open market in order to devise a solution to the difficulties encountered in the provision of workforce skills.

The widespread introduction of competition policy (Hilmer et al. 1993; Harper et al. 2015) and adopting NPM as government orthodoxy made the introduction of markets the inevitable solution to the VET problem. By adopting the ‘development of a more diverse training market’ the Australian National Training Authority (1993, 18) gave form to the market problem paradigm as a viable entity and enabled the birth phase of the market life cycle (Miller and Friesen 1984, 1162).

This allowed governments to establish the new National Vocational Education and Training System described in the July 1992 *Commonwealth and State/Territory Heads of Agreement* (1993, 3) which continued the direction set out in the earlier market reform agreement of 1990. The intention was to ‘ensure private providers are able to be registered and their courses accredited; provide targets for private and industry provider funding; [ensure] both public and private providers are equally recognised’ (Australian National Training Authority 1993, 18). These steps facilitated the growth phase (Miller and Friesen 1984, 1162) of the VET market cycle in each state and territory.

In September 2021 3644 providers met the national standards allowing them to trade as a Registered Training Organisation (RTO); another 353 RTOs were regulated by either the Western Australia or Victorian state governments which limits their delivery to domestic students in the single jurisdiction (Department of Education 2021). This level of regulatory activity demonstrates successful policy implementation, i.e., increased choice from a broad range of RTOs, because ‘Australia also has significantly more VET providers than comparable overseas markets’ (mpconsulting 2020, 15).

The Australian public policy enthusiasm for multiple suppliers competing to provide technical and vocational skills for businesses, employers and individuals has not waned since first endorsed. Responding to the COVID-19 pandemic, state, territory and federal government leaders signed the *Heads of Agreement for Skills Reform*; one of its nine priorities is ‘supporting a viable and robust system of public, private and not for profit providers, with **contestability** (original emphasis) in VET markets, to ensure high quality training and student choice’ (Commonwealth of Australia 2020, 2).

This heads of agreement reiterates more than 30 years’ bi-partisan and multi-jurisdictional support for contestable VET markets. This consensus gives effect to the political reality that ‘VET policy is a shared responsibility between the Commonwealth and the states and territories’ (Department of Prime Minister and Cabinet 2014, 11). ‘This has led to a national training system operating with consistent qualifications recognised across borders while at the same time allowing for the diversity of eight different approaches at the local level’ (Department of Prime Minister and Cabinet 2014, 11).

Internationally, federated models for the provision of VET are not uncommon. The Canadian provinces exert almost total control over the provision of VET (Cowin 2007), while in Switzerland both cantonal and federal approval for practical training standards is required (Deissinger and Gonon 2021, 5). Rather than being subject to national regulations, American and Malaysian states have the freedom to adopt their preferred vocational delivery models (Lanford et al. 2019, 2).

The evolution of Australia’s mechanisms for federal-state cooperation has been described as an example of ‘executive federalism’ which facilitates policy development characterised by long periods of stability with only incremental change because officials share a common perception of what expert knowledge is and who the experts are (Ryan 2011, 7). This policy continuity invites an exploration of how the two main drivers of VET marketisation, user choice and multiple providers, have changed over the decades and the outcomes they have produced. Clearly, the consequences of policy consistency, more choice and increased types of providers have yet to produce a perfect result. Significant recurrent problems attributed to the VET sector include low completion rates, endemic national skills shortages, poorly targeted government spending and perceptions of low-quality training (Department of Prime Minister and Cabinet 2014, 10–11).

The original 1990 policy decisions focused upon increasing choice from a broader range of providers in the market (i.e., rather than upon students, occupations or employers). Because they have exhibited a much more rapid response to introducing new providers and have been historically under-researched, this paper examines selected characteristics of RTOs headquartered in Australia’s five smallest VET markets. Provider diversity, regional subtleties and the impact of legislative, regulatory and funding changes can be more immediately recognised in these smaller markets when compared to the remaining three larger states. For example, New South Wales, took almost three years (Department of Education and Communities 2011) to implement the market-driven skills reforms that had been agreed with the national government. On the other hand, South Australia took a matter of months (South Australia Government 2010). Similarity of market size also allows for more nuanced comparisons and contrasts of changes as the market life cycle progresses.

By way of context, these five jurisdictions occupy almost two-thirds of the Australian landmass (Geoscience Australia 2021) and contain about 20% of the national population. In March 2021 Western Australia (WA) had 2.68 million residents; South Australia (SA) 1.77 million; Tasmania (TAS) 542,000; the Australian Capital Territory (ACT) 432,000 and the Northern Territory (NT) 247,000 (Australian Bureau of Statistics 2021). In 2020 Australia had 3.94 million students enrolled in VET; the three large states had 3.27 million while these five jurisdictions had 0.81 million students—just over 20% (National Centre for Vocational Education Research 2021b). Student numbers were distributed in line with population size apart from the ACT having more VET enrolments than Tasmania. Providers were similarly distributed with 19% of the nation’s current RTOs headquartered in these states and territories.

Using the methodology described in the next section, this examination of policy implementation makes novel use of publicly accessible statistics to cross-reference historical student enrolment patterns against corporate information held by statutory bodies that regulate VET providers. Data interpretation gives additional indications of how the process of creation and maintenance of training markets has progressed and supplies further quantitative information for analysing the outcomes produced by the realisation of VET markets by state, territory and federal governments.

## Methods

Because state and territory governments are ‘primarily responsible for delivering training and managing their training markets’ (Productivity Commission 2020, 159), this study adopts this perspective that each jurisdiction has its own training marketplace. However, it is recognised that more detailed analytic units such as the ‘7874 unique VET markets’ identified in the national skills and workforce agreement review (Productivity Commission 2020, 91) can be generated from different treatments of data sets. While potentially promising for future localised research, these units of analysis have no longitudinal history in the VET sector, rendering them of limited use when making long-term comparisons of market life cycles.

The National Centre for Vocational Education and Research (NCVER) curates and makes publicly available very large VET specific data sets regarding students, RTOs and qualifications studied in a variety of analytic formats that pre-date the introduction of VET markets (for example, National Centre for Vocational Education Research 2021a). These longitudinal reports have been used to calculate market shares at a particular point in time and qualifications offered in each jurisdiction. All RTOs are legally required to report nationally consistent and standardised data to the NCVER (National Centre for Vocational Education Research 2016).

All providers of Nationally Recognised Training must be a Registered Training Organisation. Those that meet the legislated standards (Australian Skills Quality Authority 2019) are recorded in the National Register of VET, a searchable online data base, along with their scope of the qualifications they have been approved to deliver/assess (Department of Education 2021). Relevant business details such as their headquarters address, regulator and initial date of registration are also listed. Each RTO must have an Australian Business Number (ABN) which identifies its type of business entity, tax status and corporate regulator. The Australian Securities and Investments Commission (ASIC) regulates for-profit businesses while the Australian Charities and Not-for-profits Commission (ACNC) oversees not-for-profit organisations (Australian Business Register 2018). ABNs were mandated in 1999 and their date of issue gives some indication of how long a business has operated.

At the end of financial year 2020–21 reports listing the current RTOs headquartered in each of the five states and territories were generated from the national register. Total VET activity reports show that these RTOs primarily operate in their local market and that interstate provision of training is generally in net balance (National Centre for Vocational Education Research 2021b). Following the initial policy logic that a wide range of providers would furnish the foundations for choice; the following RTO characteristics held by regulators were identified in order to determine the diversity of VET market providers:

- Entity unit: ABNs assign a business type to each RTO which facilitates grouping into for-profit ASIC regulated businesses or not-for-profit ACNC registered entities.
- Regulator and year of initial registration: each of these five jurisdictions’ RTOs were regulated by the national Australian Skills Quality Authority except for the 186 registered with the WA Training Accreditation Council.
- Initial active ABN year: indicates when the business commenced trading but not necessarily when it became an RTO.

## Results

This section presents the information that has been extracted from the various regulatory sources described above. Entity types give some indication of the size and reach of the RTO. For example, sole traders are likely to offer specialist training in a limited geographical or occupational area. This category also is closely aligned with the profit or not for profit nature of the business. The RTO status and ABN registration data can be used as guides as to how long an RTO has operated or undergone a major change as well as its corporate regulator. Qualifications and market shares give some indications of what choices are available to students and the range of providers that are trading in the market at particular dates which allows for comparative analysis of changes in providers over time. These factors will be interpreted in next section.

### Business entity types

Based on ABN records RTOs belong to one of eight business entity types. The relative proportion of each type is similar in all five markets with private companies making up more than half followed in descending order by other incorporated entities, government entities, public companies, discretionary trading trusts, family partnerships, fixed unit trusts and, with less than 2%, sole traders. Not all entity types are active in each market, e.g., the ACT lacks family partnerships and fixed unit trusts.

Other incorporated entities make up a large majority of RTOs subject to ACNC regulation followed by public companies then private companies. Government entities and the ACNC-registered businesses comprise the not-for-profit group of VET providers representing between WA’s 21% and the NT’s 40% of RTOs in their respective markets. Businesses from seven entities, excluding government entities, comprise the for-profit sector that rely on ASIC regulation. On average across the five jurisdictions, three-quarters of RTOs are for-profit entities ranging from a high of 80% in SA to 60% in the NT.

Table 1 presents details of regulatory groupings and the numbers of RTOs that have ever been entered onto the National Register of VET. About 50% of providers have left the markets for reasons including having voluntarily withdrawn, regulatory action to cancel registration or being non-current (Department of Education 2021). For example, WA reports that prior to the establishment of the national regulator, it regulated 508 RTOs in June 2009 in a pattern of increasing registrations that were consistently growing at about 10% per year (Training Accreditation Council Western Australia 2009, 13). Fifty% of WA RTOs ever registered have ceased trading in that market and there are currently 120 fewer registered providers than in 2009.

**Table 1:** RTO registration/regulation summary for each jurisdiction

Jurisdiction	Current RTOs	ACNC regulated	ASIC regulated	Government entities	RTOs registered > 1992
WA	388	51 (13%)	305 (79%)	32 (8%)	783
SA	176	25 (14%)	140 (80%)	11 (6%)	360
ACT	90	20 (22%)	60 (67%)	10 (11%)	165
TAS	57	11 (19%)	41 (72%)	5 (9%)	134
NT	47	14 (29%)	28 (60%)	5 (11%)	83
Total	758	121 (16%)	574 (76%)	63 (8%)	1525

### Registered training organisation status

The National Register reports the year in which an existing RTO business entity was initially listed. There are some potentially misleading dates, particularly for the public providers that are mostly treated as other incorporated entities for ABN purposes and reflect changes to state and territory governments’ organisational arrangements for their TAFEs. For example, in Tasmania the initial registration for TasTAFE is recorded as 2009 despite predecessor TAFE institutions having been in existence for decades. The 2009 entry indicates when the Tasmanian Polytechnic and the Skills Institute began delivery (Simmons 2012, 6). The RTO registration was subsequently transferred to a new entity, TasTAFE (Hansard 2013), which acquired a new ABN in 2013. Since VET markets were created, and except for the ACT, each jurisdiction has, at least once, significantly altered the organisational structure of the main public providers resulting in new initial registration dates.

Table 2 groups the original registration year for the current RTOs in each VET market. The earliest recorded date is 1992 in the NT and there are entries for every year to 2021 in one or more of the other jurisdictions (Department of Education 2021). Cumulatively, almost two-thirds of providers have been active in these five VET markets for more than ten years with 40% longer than 16 years. In addition, the pattern of initial registrations is similar in each market with a large proportion of providers entering prior to 2005, a dip in registrations in the 2010–14 period and an increase in the most recent years. The exception is SA’s increase in RTOs in 2010–14 which was not evident in the following period. The 2010 introduction of the *Skills for All* policy and funding reform had a specific goal of increasing private providers which facilitated early market entry (South Australia Government 2010, 22–23).

**Table 2:** Initial registration year as a Registered Training Organisation

Jurisdiction	RTO </= 2004	RTO 2005–09	RTO 2010–14	RTO 2015–21
WA	140 (36%)	94 (24%)	74 (19%)	81 (21%)
SA	80 (46%)	41 (23%)	30 (17%)	25 (14%)
ACT	36 (42%)	24 (27%)	12 (13%)	16 (18%)
TAS	24 (42%)	12 (21%)	9 (16%)	12 (21%)
NT	24 (51%)	11 (23%)	5 (11%)	7 (15%)
Total	304 (40%)	181 (24%)	140 (18%)	141 (18%)

In the not-for-profit group, 112 of the 121 business entities have been RTOs for longer than 13 years and only one new provider has entered any of the markets since 2018, while the last SA non-profit entrant was registered in 2013. This prompts two observations—this portion of the market is very stable and/or many not-for-profits have found the VET market unattractive—these non-exclusive possibilities will be explored in the discussion section.

Table 3 indicates the year in which the business entity registered for an Australian Business Number. An ABN is required to conduct business for certain tax and other purposes; the online acquisition process requires a few minutes and the payment of a small fee. ABNs are easily transferrable and the ASIC (2018) database indicates that numerous for-profit RTOs have shared their ABN across multiple trading names.

**Table 3:** Initial registration year for an Australian Business Number for current RTOs

Jurisdiction	ABN </= 2004	ABN 2005–09	ABN 2010–14	ABN 2015–21
WA	160 (41%)	81 (21%)	92 (24%)	55 (14%)
SA	98 (57%)	38 (21%)	17 (10%)	23 (12%)
ACT	52 (58%)	15 (17%)	11 (12%)	12 (13%)
TAS	34 (60%)	4 (7%)	12 (21%)	7 (12%)
NT	28 (60%)	7 (15%)	9 (19%)	3 (6%)
Total	372 (49%)	145 (19%)	141 (19%)	100 (13%)

As with RTO initial registrations, the ABNs of the providers describe a similar market participation with one-half of all current RTOs having been issued with an ABN more than 16 years ago and nearly 60% operating for over 10 years. This indicates that these businesses are successfully trading. More active ABNs (68) were issued than RTOs registered up until the end of 2004 suggesting that several existing businesses were not involved in training delivery in that time period but have since become an RTO. This is confirmed with 36 more RTOs added to the national register than ABNs issued between 2005 and 2009; a pattern which is repeated between 2015 and 2021.

Western Australia had the largest percentage of RTO registrations as well as the largest percentage of ABNs issued in the period 2005–21 implying that having its own registration authority positively assisted with increasing the range of providers in their market based on changed VET regulatory legislation in 2009 (Training Accreditation Council Western Australia 2009, 4). The numbers of newly registered RTOs corresponded with the issuance of ABNs between 2010 and 2014 when all five markets are added together.

In the ACNC-regulated not-for-profit sector 102 ABNs were issued more than 20 years ago out of the 121 RTOs that are currently registered. The last ABN issued in any of the five markets was acquired by an Indigenous association the NT in 2017. No ABNs have been issued for a not-for-profit RTO in Tasmania since 2003. These observations align with the interpretation of stability in the not-for-profit market sector indicated by RTO registration dates, while also indicating that VET markets are apparently no longer attractive to new potential not-for-profit providers. These are characteristics of mature markets in which growth is no longer occurring, competition for the same group of customers is more intense and effective financial control becomes a major focus (Miller and Friesen 1984, 1172).

### Qualification diversity

Total student enrolments in nationally recognised training products, regardless of funding source, show that each of the five markets offer very similar qualifications choices to students and employers (National Centre for Vocational Education Research 2021b). Non-training package qualifications; Community Services; Business Services; Tourism, Travel and Hospitality; Automotive Industry; Electrotechnology; Health; Construction, Plumbing and Integrated Services have the most student enrolments in the top 15 training package groups (out of 51) in all five VET markets. Next were Property Services; Metal and Engineering and finally, Agriculture, Horticulture and Conservation which are in the top 15 training product enrolments in four of the five markets. Qualifications drawn from 11 training packages dominate the product offerings.

An average of 85% of all students in these five markets are enrolled in the top 15 training package qualifications; ranging from 82% in the NT to 91% in WA (National Centre for Vocational Education Research 2021b). Regional occupational profiles can explain qualification differences between the VET markets. For example, the ACT is the geographically tiny administrative and bureaucratic seat of national government. Therefore, it is unsurprising that Public Services; Retail Services and the Information, Communications and Technology training packages have greater market share when compared to the higher levels of employment in primary industries and mining found in WA and the NT (National Centre for Vocational Education Research 2021b).

### Market share

Another aspect of these five training markets is explored before moving onto the interpretation and discussion—changes in market share by funding source. Prior to the introduction of Total VET Activity reporting in 2014 (National Centre for Vocational Education Research 2015), training funded from other than public sources was not reported in a consistent format, rendering it invisible to policy makers.

However, comparable data sets for government-funded VET in three major provider groups exist from 1996 onwards which allows for partial longitudinal tracking of VET market public policy implementation. Two of the provider types are generally in the not-for-profit sector; ‘TAFE and other government providers’ (TAFE) and ‘community education providers’ (CEP) while the for-profit RTOs are grouped as ‘other providers’ (OP) (National Centre for Vocational Education Research 2021a, table ten). Table 4 shows the  percentage of state and territory market share by provider type and the decline of the government-funded market size from the highest point to 2020. Calculations are based on the number of students enrolled during the year in each of the three RTO groups as reported in the *Historical time series of government-funded vocational education and training in Australia 1981–2020* (National Centre for Vocational Education Research 2021a, table ten).

**Table 4:** % market share in 2020 and reduction of government-funded market participants

Provider type	TAFE (%)	CEPs (%)	OPs (%)	Market size (%)
WA	69	~ 1	30	− 27
SA	60	3	37	− 44
ACT	63	0	37	− 30
TAS	51	0	49	− 30
NT	63	0	37	− 29
Average	61.2	0.8	38	− 32

Each of the five jurisdictions exhibits similar market share trends in the period 1996–2020. The timing and magnitude of changes differs in each jurisdiction reflecting local responsiveness to policy and program alterations. Overall, the 2020 government-funded portion of the market has declined from its highest point by about one-third which placed significant financial strain on those RTOs that derive the bulk of their income from public funding. This funding pattern precedes the decline phase of the market life cycle as profitability drops, markets dry up and the focus on survival comes at the expense of innovation (Miller and Friesen 1984, 1162).

This likely explains why CEPs and other not-for-profit providers have left the markets and the lack of new entrants from this group. The other clear pattern is a shift of market share from TAFEs and CEPs to Other Providers. For example, in 1996 Tasmanian OPs held 8% and TAFE had 90% of market share compared to 2020’s near even split (National Centre for Vocational Education Research 2021a, table ten). The past five years shows a very stable distribution between not-for-profit and for-profit providers in each government-funded market. This stasis suggests that the maturity phase of life cycle has been achieved. ‘Devoted attempts are made to arrange for a stable, negotiated environment by fixing prices and lobbying with government’ (Miller and Friesen 1984, 1171–1172).

The most recently available total VET activity reports (i.e., funded from all onshore sources) of the student enrolment market share for each provider type in the five combined markets presents a very different pattern compared to government-funding only (National Centre for Vocational Education Research 2021b). Between 2016 and 2020 the combined enrolments in the five markets declined from 839,000 to 808,000 students representing only a four% reduction compared to a 10% decrease in government-only funding, continuing the trend of reduced public funding that had commenced well prior to 2016. Apart from OPs, which grew student numbers by four%, the other provider types show an absolute decrease in enrolments with CEPs losing seven% and TAFEs shedding 16% of total student enrolments in this four-year period.

In 2020 for-profit OPs had 68% of the combined five markets’ share, the five TAFE systems enrolled 20% and CEPs had 17%. This number totals more than 100% because significant numbers of students are simultaneously enrolled with more than one provider type. Since 2016 nearly identical distributions of market shares between the three reported provider types are evident in each state and territory, again suggesting maturity of the marketplace.

## Interpretation

These five VET markets closely resemble each other based on the specific provider characteristics extracted from the large data sets. WA’s retention of its own RTO regulator is the major structural difference observed. For-profit RTOs represent 76% of the providers averaged across the five markets and they operate as one of seven business entity types, most commonly private companies. The markets can be characterised as open because about 50% of all providers ever registered have ceased trading while there has been a steady flow of mostly for-profit new entrants to a shrinking market attributed to reduced government VET funding (Joyce 2019, 27–28). The open nature of the market is further confirmed by the ABN issuance dates which indicate that a significant number of existing companies later registered themselves as RTOs in order to enter these VET markets.

Another shared characteristic of the five VET markets is that a large number of RTOs have continuously traded for long periods of time. This is particularly evident in the not-for-profit segment reflecting the dominance of well-established charities, social enterprises and government agencies/statutory authorities. Few not-for-profit providers are entering the VET market in any of the jurisdictions suggesting that increased regulation, shrinking student numbers and reduced funding render an RTO business case marginal or unviable in the mature markets.

The increase of for-profit providers, in contrast, implies their cost base is lower and they may also specialise in offering qualifications that generate a high gross profit margin (Gittins 2017). These traits are also indicators of mature markets where ‘efficiency seems to become a substitute for innovation and this requires effective financial controls’ (Miller and Friesen 1984, 1172). There has been a clear shift of market share from the not-for profit providers to profit-making RTOs although this pattern has stabilised in recent years. This movement is demonstrated in both government-funded training and total VET activity measurements. The COVID-19 pandemic has intensified some social and economic trends and the VET market is no exception. The not-for-profit adult and community education providers lost 20.7% of their enrolments between 2019 and 2020, ‘the largest drop in recent history’, compared to TAFE providers increasing enrolments by 2.5% due to new pandemic-induced government-funded programs and apprenticeship wage subsidies while for-profit RTOs declined 6.6% mirroring the total student reduction of 6.8% (Community Colleges Australia 2021, 3).

Student enrolments are heavily concentrated in a relatively small range of qualifications. This similarity in all five jurisdictions likely explains why provider types are also becoming more concentrated. Any differences between the products being sold in the various VET markets reflect the major businesses and industries in each state or territory. It has been shown that shifts in market share, entry and exit of providers and student enrolment numbers and patterns can be correlated with changes in government policies and programs. This demonstrates well-established market-driven responsiveness to changed trading conditions.

Over the years there have been proposals for the states and territories to relinquish control of their VET markets to the national government in order to achieve a single national VET market. The first serious attempt occurred when Prime Minister Keating (1992, 56) offered ‘to assume full funding responsibility for TAFE and vocational education and training’ in his *One Nation* economic statement in order ‘to take forward the national agenda for training reform’. Keating’s offer was firmly rejected by the states and territories, although the idea of Commonwealth control of VET is regularly revisited in the name of national consistency, needs and priorities (Department of Prime Minister and Cabinet 2014, 12; Joyce 2019, 73). The analysis of these five markets demonstrates very high levels of uniformity in terms of choice of provider types, contestability for public funds, training products on offer and market structure. These similarities demonstrate that even with distributed responsibilities, national market policies have been successfully achieved while still incorporating the priorities of each state or territory.

## Discussion

Despite decades-long, broad-based political support for the use of regulated markets to deliver VET in Australia, there remain persistent critics who argue against the marketisation of education and training, thereby ensuring that the policy remains contested (Quiggin 2017, 2019; Wheelahan and Moodie 2016; Wheelahan 2018; Buchanan et al. 2020). Rather than joining the debate over market versus non-market approaches to the delivery of government services (for example, Wolf 1993, 1–15) this analysis explores what statistical sources tell us about the enactment of this long-standing public policy. Interrogating substantial data sets establishes that the policy intention to increase choice of VET market providers was successfully realised in Australia’s complex political and bureaucratic federation.

In keeping with the basic features of NPM, there has been increased VET regulation in response providers who seek dubious financial advantages or offer low quality qualifications when governments design and intervene in the markets. Some of these poor VET-related behaviours have included rorting overseas student provisions to facilitate a means to gain permanent residency (Baird 2010), offering unduly short courses (Australian Skills Quality Authority 2017) and causing the massive conversion of public funds into private profit by exploiting the VET FEE-HELP student loans scheme which burdened students with personal debts and useless enrolments (The Auditor-General 2016; Yu and Oliver 2015). The outsourcing of responsibility for policy failure typically blames poor contract specification (Keating 2004, 98–100). For example, the Productivity Commission (2020, 23) believes that ‘the rorting associated with VET FEE-HELP stemmed from flaws in the design and implementation of the program’ and remediation required ‘substantially strengthened integrity measures and tighter provider eligibility criteria’, i.e., increased regulation of the VET market.

The research findings reported in this article indicate that the original policy intention to increase the numbers and diversity of RTOs to facilitate user choice exemplifies successful policy implementation on the part of state, territory and federal governments. ‘Given that competition has been a feature of the VET system since the 1990s, VET markets are now well established’ (Productivity Commission 2020, 122). The effects of increasing this choice of provider are equally evident because VET sector cost efficiencies have been achieved (Noonan 2016, 13–16) and an increasingly regulated marketisation of former government monopolies has also occurred (Wheelahan 2017).

The VET markets of the five jurisdictions offer a heavily concentrated range of products, show very similar distributions and numbers of providers linked to population size, are extensively regulated and are supported by government-funding; for most purposes they are like each other.

In light of Polanyi’s (2001, 146) proposition that markets are not the natural order and that ‘the road to free markets was opened and kept open by an enormous increase in continuous, centrally organised and controlled interventionism [by the state]’, it has to be considered that the various Australian VET markets have matured and are stabilising around an equilibrium point enabled by three decades of government policy consistency.

This research finds unequivocal evidence that the diversity of providers and qualifications on offer in these five VET markets are diminishing, thereby limiting choices available to students and government procurers. The observation that community education and not-for-profit providers have left VET markets is further supported by comparing two studies. The first national survey of not-for-profit social enterprises found that 42% listed education and training as their main area of operations (Barraket et al. 2010, 21). Six years later a similar social enterprise survey found that there had been a mass shift out of this business area with only 18% reporting education and training as their principal activity (Barraket et al. 2016, 16). The states and territories were further opening their markets to for-profit providers during this time period to implement provisions in the National Agreement for Skills and Workforce Development (Council of Australian Governments 2008, 6).

The creation of VET sector markets occurred on the basis that the absence of choice of provider was the problem requiring resolution. The data explicitly demonstrates that successful public policy implementation of increased choice has extracted market-driven benefits out of the National Training System as originally intended. Analysis of major data bases also indicates that after more than three decades the limits of marketisation have been reached due to a limited choice of training products on offer and a clear pattern of reduced diversity of providers. Adult and community RTOs are leaving the sector and similar entities are no longer entering the markets. The combination of increased regulatory barriers to entry, a shrinking market and reduced levels of funding makes profitability unsustainable for all except the leanest or most highly specialised for-profit providers or publicly funded TAFEs. Each of these indicates that these Australian VET markets have reached the end of the maturity phase of their life cycles and are entering the decline phase.

Torfing et al. (2020, 16), describe a similar pattern in England; ‘new and emerging governance paradigms initially tend to produce gains and benefits, after a period of consolidation, their marginal return to scale slowly begins to decline and the unintended negative consequences become increasingly apparent’. Their large-scale evaluation of 30 years’ use of NPM concludes that markets eventually achieved the exact opposite of what was originally promised. The repetitious application of marketisation blocked the consideration of other, more promising, reforms to the provision of public services (Torfing et al. 2020, 66–68).

This policy myopia is evident in VET markets. It is also suggested that the business logic that underpins NPM has led to the ‘loss of public service [policy] capability’ and replaced it with ‘a corporate management model at risk of exhausting the benefits of its paradigm’ (Davis 2021, 10). Perhaps more importantly, Loasby (2007, 1750) argues that there is ‘no other principle so dangerous as that of one best way, because insisting on one way is almost certain to prevent anyone finding the best, or even a better way’.

30 years after the acceptance and implementation of market policies, it has become evident as to which parts of the highly diverse VET sector benefit from the discipline of the market and those areas that are more suited to direct government interventions as evidenced by those RTOs that have successfully traded over long periods of time. Clearly state and territory governments want to retain their capability to intervene directly in their VET markets on the supply-side, rather solely relying on their ability to influence market-driven behaviours. Despite significant VET expenditure reductions by these governments (Joyce 2019, 28), none has chosen to follow the Productivity Commission’s (2012, 42) suggestion to disband government-operated TAFE institutions. Rather states and territories have been willing to reduce or even eliminate funding a market share for community education providers. The interpreted data demonstrates that the three decades of policy stability and successful implementation have delivered the outcomes originally expected from VET markets, but it also exposes perverse and unintended consequences that are appearing as they move towards the end of the market life cycle.

What other policy options have emerged that might re-energise the sector? Stanley (2017, 3) argues for optimisation because competition policies rarely produce optimal results. Competition’s intention is to increase contestation rather than deliver what is best for consumers and society. Rather, optimisation achieved by integrating competition and cooperation should be the public policy goal and ‘some industries should be managed as systems’ which are networks of ‘interdependent components that are managed to accomplish the aims of the system’ (Stanley 2017, 8).

Some industries need to be managed as systems by an overseeing agency and ‘will benefit from a triangular, cooperative relationship between government, industry and competitors’ which will ‘help industries to expand their market, lower prices and improve quality by minimising waste, inefficiencies and duplication’ (Stanley 2017, 16). Regulating the large number of providers trading in the Australian VET market comes at a significant cost; the national regulator spent almost $44 million in 2020–21 (Australian Skills Quality Authority 2021, 105) in addition to the expenditure by the WA and Victorian regulators. There are also vast amounts of duplication of enrolment/reporting systems, facilities, advertising and stand-alone digital technologies incurred by the nearly 4000 providers that meet the requirements to be an RTO. Given the diversity, complexity and unresolved problems found in Australian VET, system optimisation deserves further consideration rather than continuously returning to the familiar and comfortable option of contestable markets.

While open market competition based on provider choice has dominated economic models of western liberal democracies since the 1980s, other economic theories potentially relevant to VET markets have assumed greater prominence in recent years. Public Value Management (Torfing et al. 2020, 105–124; Mazzucato and Ryan-Collins 2019) is a paradigm of public sector management that also facilitates system optimisation because it accepts diversity and rejects the dualisms of the market-nonmarket binary. Public Value Management reminds us that governments exist to serve their communities by creating public value (Torfing et al. 2020, 105–107).

‘Public value is both validated and produced by a broad range of public and private actors engaged in public dialogue and collaborative interaction’ (Torfing et al. 2020, 31). Mazzucato and Ryan-Collins (2019, 14) reject the notion of market failure and its supposition of community service obligations to justify public provision. Public value is created by having the public sector set the direction and public purpose in ways that enable private and public actors to co-design, collaborate and innovate to solve societal problems. These more contemporary economic proposals could be used to shift the VET markets back to the revival life cycle phase characterised by product diversification, high levels of innovation and increased use of formal coordinating controls (Miller and Friesen 1984, 1178).

## Conclusion

Using a non-traditional method of linking longitudinal enrolment and regulatory data demonstrates that the Australian public policy intention of increasing the numbers and types of registered training organisations competing in markets for the delivery of vocational education and training products was successfully implemented long ago. The goal of a nationally consistent and highly regulated VET marketplace has, in effect, been achieved with a similar suite of qualifications on offer from an analogous range of providers in the five states and territories included in this research project. Each jurisdiction has also retained the capability to create and intervene in their market in ways that meet local priorities in terms of who can train in specific occupations. Clearly there are market forces at work with gains in efficiency in some fields, providers entering and exiting the markets and regulators monitoring national standards and quality.

The quantitative evidence also demonstrates that in more recent years the market structures have stabilised, and that not-for-profit and community education providers are leaving the market. Similarly, the range of products on offer to students has become heavily concentrated in just a few occupational groupings. Both serve to reduce choice in the now mature markets and could be factors that are driving students away from the sector.

This result delivers the exact opposite of the original policy intention to realise the benefits of increased choice to grow VET enrolments. It is arguable that after 30 years of using the imported New Public Management paradigm to guide VET policy that all the first-mover benefits have been realised and it is now time to explore more contemporary economic theorisations on how to create and maintain public value in an optimised national training system. There is an imperative to embrace the social, occupational and economic knowledges of the 2020s rather than uncritically apply market-based solutions from the 1980s whose origins lie in the 1950s.

However, before the way can be cleared to better understand if system optimisation through public value management styles can be applied to the mature Australian VET markets, a major policy reorientation is required. Markets were successfully implemented but their age and limitations are becoming increasingly visible as they approach the end of their life cycle. VET needs to be re-problematised to assist the governments that created the markets to consider more modern means of vocational skills acquisition and transfer to meet the requirements of the contemporary Australian economy by harnessing government and private sectors to create and increase public value by optimising the benefits of both market and non-market contributions to society.

## Data Availability

The datasets used and/or analysed during the current study are fully referenced, publicly accessible and are available from the corresponding author on reasonable request.
